# *Clostridium butyricum* regulates intestinal barrier function via trek1 to improve behavioral abnormalities in mice with autism spectrum disorder

**DOI:** 10.1186/s13578-024-01278-6

**Published:** 2024-07-21

**Authors:** Simeng Liu, Huayuan Xi, Xia Xue, Xiangdong Sun, Huang Huang, Dongjun Fu, Yang Mi, Yongzheng He, Pingchang Yang, Youcai Tang, Pengyuan Zheng

**Affiliations:** 1 Marshall B. J. Medical Research Center, Zhengzhou University, Zhengzhou, 450052 Henan China; 2 Xiangyu Medical CO., LTD, Anyang, 456300 Henan China; 3 Department of Gastroenterology, the Fifth Affiliated Hospital of Zhengzhou University, Zhengzhou, 450052 China; 4 Modern Research Center for Traditional Chinese Medicine, School of Chinese Materia Medica, Beijing University of Chinese Medicine, Beijing, 100029 China; 5 Brain Body Institute, McMaster University, Hamilton, ON Canada; 6 Department of Pediatrics, the Fifth Affiliated Hospital of Zhengzhou University, Zhengzhou, 450052 China

**Keywords:** Autism spectrum disorder, Intestinal barrier function, Trek1, Gut microbiota, *Clostridium butyricum*

## Abstract

**Background:**

Autism Spectrum Disorder (ASD) is a complex neurodevelopmental disorder that has been found to be associated with dysregulation of gastrointestinal functions and gut microbial homeostasis (the so-called “gut-brain axis”). ASD is often accompanied by poor performances in social interaction and repetitive behaviors. Studies on the gut-brain axis provide novel insights and candidate targets for ASD therapeutics and diagnosis. Based on the ASD mice model, this work aims to reveal the mechanisms behind the interaction of intestinal barrier function and probiotics in ASD mouse models.

**Results:**

We found an altered intestinal barrier in both BTBR T^+^ Itpr3^tf^/J **(**BTBR) and valproic acid (VPA) mice, including increased intestinal permeability, decreased expression of intestinal tight junction proteins (claudin1, claudin3, and occludin), and increased levels of IL-6, TNF-α, and IFN-γ. Based on intestinal microbial alternation, *C. butyricum* can drive reduced expression of histone deacetylases 1 (HDAC1) and enhanced intestinal barrier function, significantly promoting behavioral abnormalities of ASD in BTBR mice. In parallel, we confirmed that *C. butyricum* was involved in the regulation of intestinal function by the Trek1 channel, indicating that it is a target of *C*. *butyricum*/butyric acid to improve intestinal barrier function in ASD mice.

**Conclusions:**

Our finding provides solid evidence for the gut microbiota involved in ASD through the brain-gut axis. In addition, the probiotics *C*. *butyricum* hold promise to improve gut health and ameliorate behavioral abnormalities associated with ASD.

**Supplementary Information:**

The online version contains supplementary material available at 10.1186/s13578-024-01278-6.

## Background

Autism spectrum disorder (ASD) is a multifactor neurodevelopmental disorder characterized by difficulties in social interaction, such as avoiding eye contact, narrowed vocabulary, and repetitive behaviors [1]. The overall prevalence of ASD around the world is about one in 100 children, according to the WHO 2023 statistical report [2]. The etiology is complex and heterogeneous among ASD cases, involving genetic and environmental factors [3]. More than 85% of people with ASD have been reported to have gastrointestinal (GI) abnormalities and dysregulated gut microbial homeostasis [4]. The previous study confirm that intestinal microbial community is different in ASD individuals compared with it from normal ones [5]. According to the hypothesis of the brain-gut axis, the gut microbiota and its metabolites play an essential role in the pathogenesis of ASD, which provides new potential therapeutics and nutritional interventions to alleviate symptoms of ASD. However, the systematic mechanism behind the brain-gut microbiota axis has not been fully elucidated.

Increasing evidence has shown that the gut microbiota contributes to neural development and function in animals [6, 7]. It affects various complex behaviors, including repetitive behaviors [8], cognitive performance [9], social communication [10], and anxiety [11]. The gut microbiota can communicate with the central nervous system (CNS) through immune, metabolic, endocrine, and neural pathways, in part due to its contributions to metabolism and immune homeostasis [12]. The gut microbiota provides the host with multiple functional components (e.g., short-chain fatty acids, serotonin, γ-aminobutyric acid), which are significant on the “microbiome-gut-brain axis” [13]. The alterations of the gut microbiota and microbial metabolites can also lead to immune dysregulation, which influences neurodevelopment and neurogenesis [14, 15]. Furthermore, the gut microbiota can be associated with ASD by influencing intestinal barrier function [16, 17]. The compromised intestinal barrier function has been linked to behavioral abnormalities, including those observed in ASD. The intestinal barrier serves as a protective barrier that regulates the passage of nutrients, toxins, and microbial metabolites between the gut lumen and the systemic circulation [18, 19]. Disruption of intestinal barrier integrity can lead to increased permeability, allowing the translocation of harmful substances and triggering immune activation [20].

This study aims to evaluate the role of the gut microbiota and its metabolites in the function of the intestinal epithelial barrier to reveal the pathogenesis of ASD. BTBR T^+^ Itpr3^tf^/J (BTBR) [21] and valproic acid (VPA) mice [22], the commonly used genetic and environmental risk factors for mouse models of ASD, were used in the present study. Understanding the interplay between the gut microbiota, intestinal barrier function, and TWIK-related potassium channel-1 (Trek1) signaling could provide valuable insights into the underlying mechanisms of ASD pathogenesis and potentially identify novel therapeutic targets for the management of symptoms related to ASD.

## Methods

### Animals and ethics statement

C57B/6J (B6J) and BTBR T^+^ Itpr3^tf^/J mice in this study were obtained from the Nanjing Institute of Biomedical Research of Nanjing University (Nanjing, China). The mice were housed in a room controlled by humidity in a 12 h light/dark cycle with free access to a standard diet and water. All animal procedures were approved by the Ethics Committee of the Fifth Affiliated Hospital of Zhengzhou University (No. 2016-1001) and carried out according to the Guidelines on the Care and Use of Animals for Scientific Purposes (National Advisory Committee for Laboratory Animal Research). All animals were anesthetized by intraperitoneal injection of 5% chloral hydrate (500 mg/kg) and then sacrificed [23].

### Study design treatment and groups

The B6J and BTBR mice were paired overnight. Pregnant B6J mice (aged 8–10 weeks) were injected i.p. on E12.5 with saline or 600 mg/kg VPA (Sigma Aldrich, USA) according to the methods described by Almeida [24]. Only male offspring were included in this study. Male offspring were randomly selected (no more than 2 offspring from the same litter) for each test. For *C. butyricum* treatment, cages between the VPA and BTBR groups were randomly selected for treatment with *C. butyricum* or vehicle, intragastric administration, every day for 21 days from 3 weeks of age. The behavioral tests were conducted and analyzed according to Hsiao [25] and Malkova [26] (supplementary file1). Mice were tested from 6 to 10 weeks of age, following this order: open field exploration, marble burying, social interaction, adult ultrasonic vocalizations, and self-grooming, with at least 5 days intervals between behavioral tests. Mice were sacrificed at 11 weeks of age.

### DNA extraction and amplification of bacterial 16S rRNA

Microbial bacterial DNA was extracted from fecal pellets from mice using a QIAamp Fast DNA Stool Mini Kit (QIAamp, California, USA) according to the manufacturer’s instructions. The V3-V4 regions of the 16S bacterial rRNA were amplified using primers (338 F: 5’-ACTCCTACGGGAGGCAGCAG-3’ and 806 R: 5’-GGACTACHVGGGTWTCTAAT-3’) in a thermocycler PCR system (GeneAmp 9700, ABI, USA). Amplicons were extracted and further purified using the AxyPrep DNA Gel Extraction Kit (Axygen Biosciences, Union City, CA, USA) and quantified with a QuantiFluorTM-ST (Promega, USA) following the manufacturer’s protocols.

### Illumina MiSeq sequencing and analysis

Sequencing data were pooled equimolarly and sequenced in pairs (2 × 300) on an Illumina MiSeq platform (Illumina, San Diego, USA) according to the standard protocols of Majorbio Bio-Pharm Technology Co. Ltd. (Shanghai, China). The raw reads were demultiplexed and quality-filtered by Trimmomatic [27]. The reads were merged by FLASH as described previously [28]. Operational Taxonomic Units (OTU) were clustered with a 97% similarity cut-off using UPARSE (version 7.1 http://drive5.com/uparse/), and chimeric sequences were identified and removed using UCHIME. The taxonomy of each 16S rRNA gene sequence was analyzed by the RDP Classifier algorithm (http://rdp.cme.msu.edu/) against the Silva (16S rRNA database (SSU123) using a confidence threshold of 70%.

### Fecal water content

Free-feeding mice were observed for 2 h in the single cage. The fecal pellets were weighted before and after drying (24 h at 105 °C) [29]. Fecal water content (%) = (wet weight − dry weight)/wet weight × 100%.

### Small-intestinal transit ratio

The test was modified on the basis of Zhou et al. and Rtibi et al. [30, 31]. Mice were fasted for 16 h (water ad libitum) prior to intragastric gavage with a 10 ml/kg suspension of 5% charcoal in 5% gum arabic the next day. After 25 min, the mice were sacrificed and the small intestine (from the gastric-pyloric junction to the ileocecal junction) was excised. Small intestinal (SI) transit ratio (%) = migration distance of the most distal end portion of the charcoal/total length of the small intestine × 100%.

### Fecal SCFA analysis

Short-chain fatty acids (SCFA) were extracted as described previously [32]. Briefly, 300 mg of stool was homogenized with 1 mL of ddH_2_O and centrifuged at 12,000 g for 10 min. The supernatant was homogenized with 100 μL concentrated HCl and subsequently extracted for 20 min using 5 mL of diethyl ether. After centrifugation (3,500 rpm, 10 min), we mixed the organic phase with 500 μL NaOH (1 M). Then it was extracted and centrifuged again. 100 μL concentrated HCl mixed with the aqueous phase and filtered through a 0.22 μm filter. The High-Performance Liquid Chromatography System (HPLC, Waters alliance e2695, Milford, USA) with UV detector (Waters 2468, Milford, USA) was performed using a Venusil ASB C_18_ (4.6 × 250 mm, 5 μm, China) set at a flow rate of 1 mL/min at 210 nm, 30 °C. The mobile phase consisted of 0.01% H_3_PO_4_ in HPLC grade water (A) and methanol (B). All samples were carried out in triplicate.

### Intestinal permeability assay

Mice were fasted for 4 h before gavage with 600 mg/kg of 4-kDa FITC-dextran (Sigma Aldrich, USA). After 4 h, serum samples were read for fluorescence intensity with a fluorescence spectrometer (Hitachi, Japan) at the excitation wavelength of 485nm and the emission wavelength of 535 nm [33].

### Protein preparation and western blot

Total proteins were extracted from colon tissue, separated on SDS–polyacrylamide gel, and transferred to a polyvinylidene fluoride membrane. After blocking with 5% skim milk for 1h, the membrane was incubated with primary antibodies overnight at 4 °C, followed by incubation with HRP-conjugated second antibodies for 1 h. Protein bands were visualized with a Pierce ECL Western Blot Substrate (Thermo Fisher, USA). The results were photographed with a ChemiDoc XRS + system (Bio-Rad, USA). The density of immune bolts was measured with Photoshop and presented as a percentage of the internal loading control β-actin. The primary antibodies and dilutions used are as follows: anti-claudin 1 (1:1000, abcam, USA), anti-claudin 3 (1:500, abcam, USA), anti-ZO-1 (1:500, abcam, USA), anti-Occludin (1:10000, abcam, USA), anti-Trek1 (1:200, Santa Cruz Biotechnology, USA), anti-HDAC1 (1:100, Santa Cruz Biotechnology, USA) or anti-β-actin (1:500, abcam, USA).

### RNA extraction and real-time PCR

Colon tissues were flushed with PBS and homogenized in TRIzolreagent (Invitrogen, USA) for RNA isolation. Reverse transcription was performed using SuperScript III reverse transcriptase (Invitrogen, USA). Real-time PCR was carried out using a 7500 real-time PCR system (Thermo Fisher, USA) with SYBR Green Master Mix according to the manufacturer’s instructions. The 2^−ΔΔCT^ method was used to calculate fold changes in gene expression with GAPDH as internal control. Please find the primers used in this study in supplementary Table 1 of supplementary file 1.

### Immunohistochemistry

Colon tissues were dissected and fixed in 10% formalin overnight, then dehydrated, embedded in paraffin, and sectioned (5-µm). The colon sections were conventional dewaxing with water and conducted according to the protocols of the Immunohistochemistry kit (Origene, China). Briefly, sections were incubated with TREK-1 antibody (1:100, Santa Cruz Biotechnology, USA) diluted in 0.05% Tween 20 with PBS for 1 h. The sections were then washed twice for 5 min in 0.05% Tween 20 with PBS and then incubated with mouse general secondary antibody (Origene, China) for 1 h. The sections were then washed as above, rinsed in water, mounted with hematoxylin (Wexin, Guangzhou, China), and examined using a confocal microscope (Leica Microsystems DMI8 DFC7000T, USA). IHC images were semiquantitated using Image J to validate the gene expression level, as previously described [34].

### Enzyme-linked immunosorbent assay

Colon tissues were weighted and homogenized on ice. After 5,000 g of centrifugation for 5 min at 4 °C, the supernatants were collected and stored for ELISA. Levels of IL-6, TNF-α, and IFN-γ were detected by ELISA following the manufacturer’s instructions (Wuhan Huamei Bioengineering Co., Ltd, China).

### Statistical analysis

Data were analyzed using SPSS 20.0 software (IBM Corp., Armonk, N.Y., USA) [35]. Differences between BTBR/VPA and the control group were accessed using the Wilcoxon rank sum test (quantitative data, unknown variance) and Dunnett's t test (quantitative data, equal variance). Data are plotted as mean ± SD by GraphPad Prism 7.0 [36]. Numeric expressions of P values were provided for each analysis, with statistical significance defined as *p* < 0.05 (*p < 0.05, **p < 0.01 and ***p < 0.001).

## Results

### The BTBR and VPA offspring display ASD-Related behavioral abnormalities

**Fig. 1:**
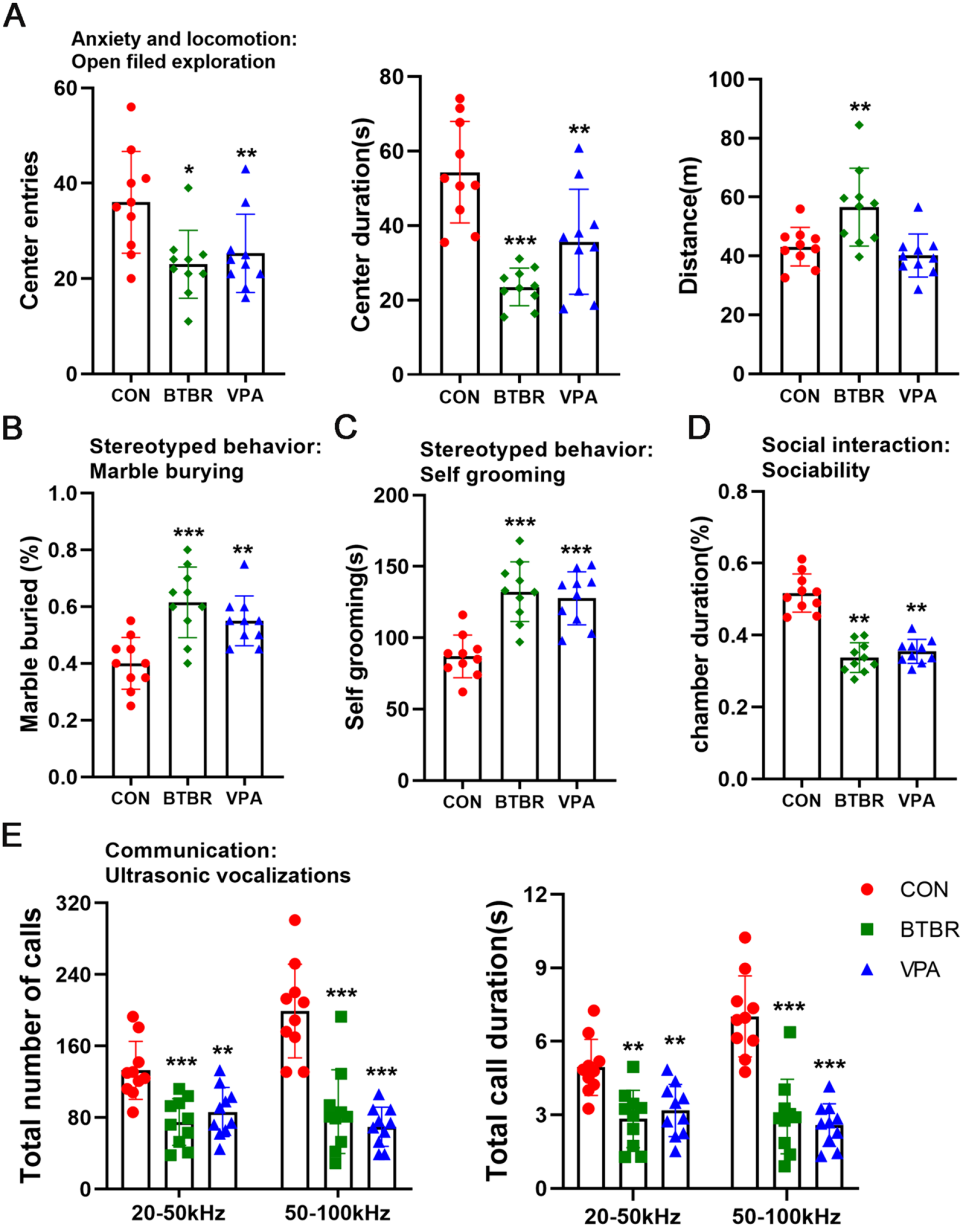
BTBR and VPA offspring display ASD-Related Behavioral Abnormalities. **A** Open field exploration test **B** Marble burying test **C** Self-grooming test **D** Social interaction test. **E** Adult ultrasonic vocalizations in a social encounter with a female. Significant differences are indicated by *p < 0.05, **p < 0.01 and ***p < 0.005. n = 10/group

Open-field exploration is widely used to measure anxiety-like locomotor behavior in rodents. The BTBR and VPA offspring showed decreased entry into the center and time spent in the center (Fig. 1A). The BTBR offspring showed an increase in total distance traveled compared to the controls, possibly due to a longer body length and heavier weights. Marble burying and self-grooming are used to evaluate repetitive and stereotypical behavior in small rodents. BTBR and VPA offspring display increased buried marble (Fig. 1B, Fig. S1A) and time spent in self-grooming (Fig. 1C) compared to the vehicle-treated B6J offspring. Furthermore, a three-chamber apparatus was used to measure ASD-related impairments in sociability [37]. The offspring of BTBR and VPA mothers exhibit a decreased preference to interact with a novel mouse over a novel object, spending less time exploring the social chamber than the controls (Fig. 1D). Finally, ultrasonic vocalizations (USVs) are used to evaluate the communication of mice. Adult males produce USVs in response to females, which is a prominent social characteristic and well correlated with the level of mating interaction. In our study, the BTBR and VPA offspring emit a reduced number and total duration of ultrasonic vocalizations (Fig. 1E, Fig. S1B), indicating communication deficits related to ASD. Together, these results suggest that the offspring of BTBR and VPA mothers exhibit cardinal symptoms of autism-related behavioral abnormalities, confirming the validity of these independent mouse models for modeling ASD-related risk factors.

### BTBR and VPA offspring display intestinal barrier dysfunction

**Fig. 2:**
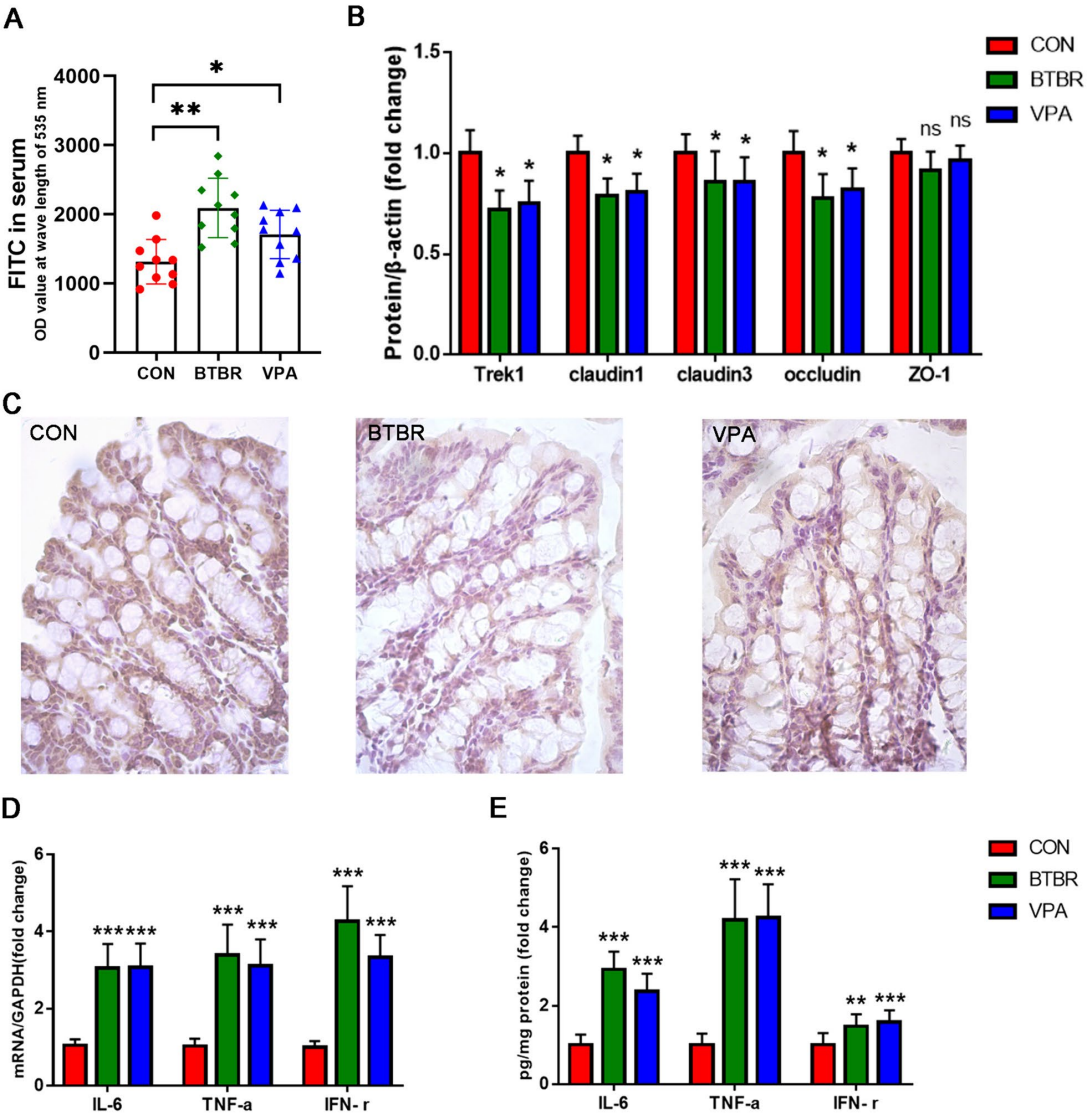
BTBR and VPA offspring display intestinal barrier dysfunction. **A** Intestinal permeability assay, measuring translocation of 4-kDa FITC-dextran from the intestinal lumen into the blood. **B** Colon protein levels of Trek1 and tight junction components relative to β-actin. **C** Immunohistochemistry staining for Trek1(400X). **D** Colon mRNA expression of cytokines relative to GAPDH. **E** Colon protein expression of cytokines. Data are normalized to controls. n = 10/group

To observe gastrointestinal abnormalities correlated with ASD in our mice models, we investigated the fecal water content and the small intestinal transit ratio. It showed that fecal water content decreased in BTBR offspring, but not in VPA offspring (Fig. S2A). No significant differences were found between the BTBR/VPA offspring and the controls in the SI transit ratio (Fig. S2B). In parallel, we found an increased translocation of FITC-dextran across the intestinal epithelium into the circulation in the offspring of BTBR and VPA (Fig. 2A). Our previous study revealed that Trek1, a two-pore domain potassium channel located in the epithelium, can also regulate intestinal barrier function [38]. According to this phenotype, the colon of BTBR and VPA offspring contains decreased expression of Trek1 and barriers forming tight junction (TJ) components (including claudin1, claudin3, and occludin). Regarding the protein level of zonula occludens-1 (ZO-1), we did not observe a significant difference between BTBR/VPA offspring and the controls (Fig. 2B). Furthermore, immunochemistry demonstrated that the expression of Trek1 in the intestinal layers was reduced in the BTBR and VPA offspring compared to controls (Fig. 2C; Fig. S3). In addition to TJs and Trek1, activation of the immune response has the potential to affect intestinal barrier integrity. Consequently, increased expression of IL-6, TNF-α and IFN-γ mRNA and proteins was observed in the colon of BTBR and VPA offspring (Fig. 2D, E). Therefore, our results confirm that deficits in intestinal barrier integrity are detectable in BTBR and VPA offspring.

In particular, we also detected intestinal permeability, colon expression of Trek1, TJ, and cytokines in 3-week-old BTBR and VPA offspring. The results were consistent with those of adult BTBR and VPA offspring. Briefly, 3-week-old BTBR and VPA offspring performed increased intestinal permeability (Fig. S4A), decreased protein expression of Trek1, claudin1, claudin3, and occludin (Fig. S4B), and increased levels of IL-6 and TNF-α mRNA (Fig. S4C). It suggests that intestinal abnormality can be found in early childhood in ASD mice.

### BTBR and VPA offspring exhibit altered intestinal microbiota and SCFA

**Fig. 3:**
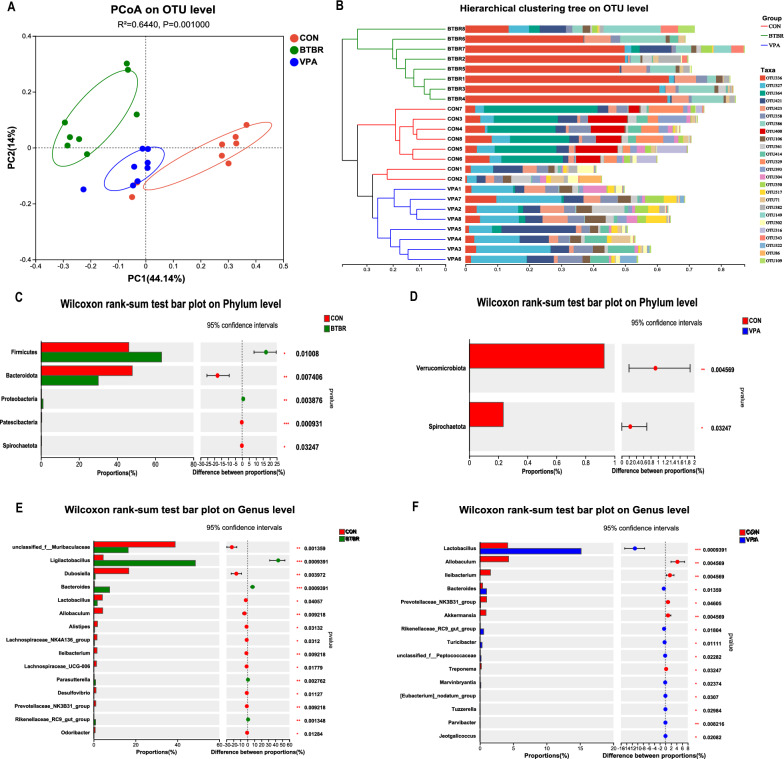
BTBR and VPA Offspring exhibit altered microbial beta diversity. **A** PCoA based on OTU level (abund_jaccard). **B** Bray_curtis based Hierarchical clustering tree on OTU level. **C**, **D** Mean relative abundances of taxa on phylum level. **E**, **F** Mean relative abundances of taxa on genus level. Significant differences are indicated by *p < 0.05, **p < 0.01 and ***p < 0.005. n = 8/group

To investigate the characteristics of the intestinal microbiota in ASD mouse models, we sequenced the hypervariable regions V3-V4 of the fecal bacterial 16S rRNA of the BTBR, VPA, and B6J offspring. Analysis of alpha diversity revealed lower species richness (Sobs, Chao, and ACE), diversity (Shannon and Simpson), and evenness (Shannoneven and Simpsoneven) in BTBR offspring (Fig. S5). However, no significant differences were observed between the control and VPA offspring regarding these indices (Fig. S5). We further performed principal component analysis (PCA) in terms of beta diversity based on all OTUs. It revealed that the bacteria of the BTBR and VPA offspring clusters apart from those of the controls (Fig. 3A). Consistent with the PCA result, hierarchical clustering analysis revealed distinct clusters that distinguished BTBR, VPA, and B6J offspring (Fig. 3B).

We evaluated the mean relative abundances of the taxa in these groups to discriminate the specific alterations of the microbiota. At the phylum level, Firmicutes and Proteobacteria were more abundant in the BTBR offspring, while Bacteroidota, Patescibacteria, and Spirochaeta were more abundant in the control offspring (Fig. 3C). We found that Verrucomicrobiota and Spirochaeta were significantly decreased in VPA offspring compared to controls (Fig. 3D). It indicated a marked change in the structure of the intestinal microbial community in BTBR offspring characterized by a higher Firmicutes/Bacteroidetes ratio (Fig. S6). At the genus level, *Ligilactobacillus* was more abundant in BTBR offspring, while *Muribaculaceae* was more abundant in control offspring (Fig. 3E, supplementary file 2). *Lactobacillus genus* was significantly increased in VPA offspring compared to controls (Fig. 3F, supplementary file 3). In particular, the genus *Bacteroides* in both BTBR and VPA showed enriched compared to the control group.

Furthermore, SCFA mainly consist of acetic, propionic, butyric, and valeric acid. In the present study, the BTBR offspring showed lower levels of fecal acetic acid, butyric acid, and valeric acid than the controls. On the contrary, the VPA offspring showed higher levels of propionic acid and lower levels of butyric acid than the vehicle-treated B6J offspring (Table 1).

**Table 1:** Content of SCFA detested by HPLC in three groups

Category	AA(ug/mL)	PPA(ug/mL)	BTA(ug/mL)	VA(ug/mL)
CON	520.90 ± 163.71	98.92 ± 15.41	1676.70 ± 257.24	10848.33 ± 1995.25
BTBR	294.56 ± 53.68**	100.10 ± 39.92	1244.88 ± 363.07**	4532.49 ± 2265.85**
VPA	635.32 ± 135.11	158.98 ± 44.73**	1300.39 ± 297.01*	10614.48 ± 1442.23

*AA* acetic acid, *PPA* propionic acid, *BTA* butyric acid, *VA* valeric acid

^*^p < 0.05 and **p < 0.01. n = 10/group

In our prior work, we also detected the composition of the gut microbiota and fecal SCFAs in children with ASD and found that lower concentration of fecal *C. butyrate* in ASD individuals. Moreover, we observed gut microbiota alternation (reduced butyrate-producing taxa and enriched valeric acid-associated bacteria) in ASD individuals, indicating modulating the gut microbiota, particularly *C. butyrate*-producing bacteria, could be a promising strategy for alternatives for the treatment of autism spectrum disorder [39].

### *Clostridium butyricum* modulates the intestinal barrier in BTBR and VPA offspring

**Fig. 4:**
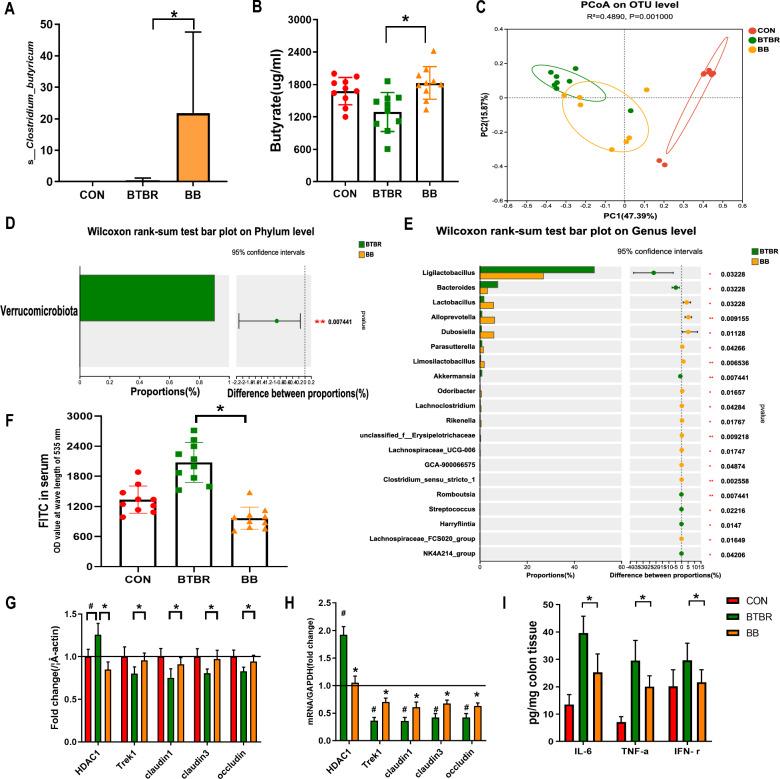
The intestinal barrier function of BTBR mice under *Clostridium butyricum* treatment. **A** The richness of *Clostridium butyricum* from fecal samples of control, BTBR, and BB mice. **B** The concentration of butyrate (ug/ml) from fecal samples of control, BTBR, and BB mice. **C** The PCoA based on OTU levels of ontrol, BTBR, and BB mice. **D** The wilcoxon rank-sum test on phylum level in BTBR mice with and without *Clostridium butyricum* treatments. **E** The wilcoxon rank-sum test on genus level in BTBR mice with and without *Clostridium butyricum* treatments. **F** The level of FITC in control, BTBR, and BB mice. **G**, **H** The expression level of tight junction proteins in control, BTBR, and BB mice. **I** The expression levels of pro-inflammatory cytokines in BTBR mice with and without *Clostridium butyricum* treatments. *p < 0.05 vs. BTBR group, ^#^*p* < 0.05 vs. CON group and **p < 0.01 vs. BTBR group

**Fig. 5:**
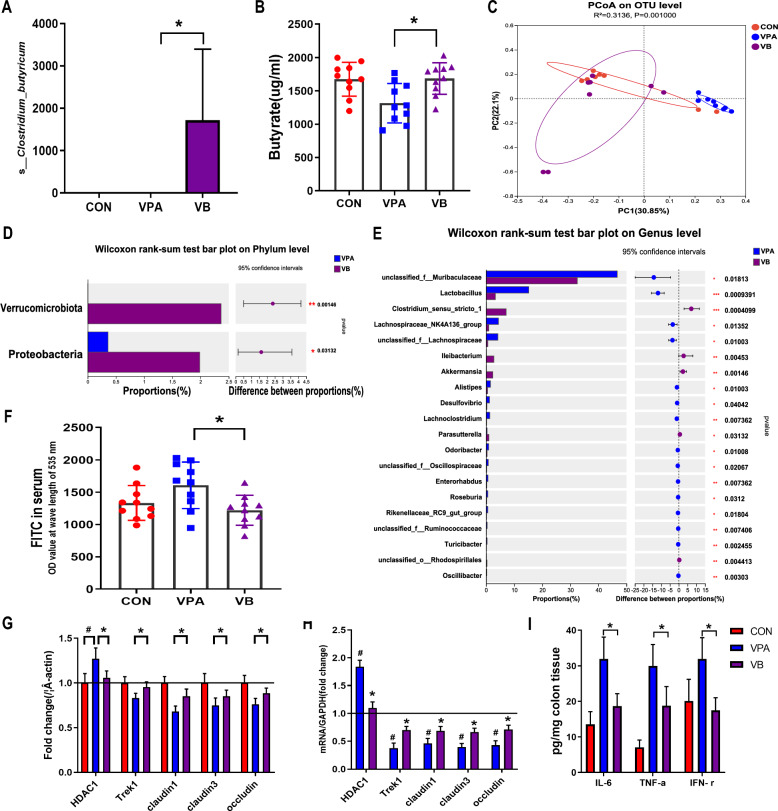
The intestinal barrier function of VPA mice under *Clostridium butyricum* treatment. **A** The richness of *Clostridium butyricum* from fecal samples of control, VPA, and VB mice. **B** The concentration of butyrate (ug/ml) from fecal samples of control, VPA, and VB mice. **C** The PCoA based on OTU levels of ontrol, VPA, and VB mice. **D** The wilcoxon rank-sum test on phylum level in VPA mice with and without *Clostridium butyricum* treatments. **E** The wilcoxon rank-sum test on genus level in VPA mice with and without *Clostridium butyricum* treatments.** F** The level of FITC in control, VPA, and VB mice. **G**, **H** The expression level of tight junction proteins in control, VPA, and VB mice.** I** The expression levels of pro-inflammatory cytokines in VPA mice with and without *Clostridium butyricum* treatments. *p < 0.05 vs. VPA group, ^#^*p* < 0.05 vs. CON group, **p < 0.01 vs. VPA group and ***p < 0.001 vs. VPA group

Mice from BTBR and VPA were treated with *C. butyricum* (BB and VB group) and their intestinal barrier function was individually evaluated (Figs. 4 and 5). In both the BB and the VB groups, the level of *C. butyricum* and butyrate increased compared to the control and untreated groups (Figs. 4A, B and 5A, B). A microbial alternation was observed in BB and VB mice (Figs. 4C, 5C), notably, the *Verrucomicrobiota* phylum showed more abundance in VB mice only (Figs. 4D, 5D). The comparison of genus and species level showed a similar pattern between mice treated with and without *C. butyricum* (Figs. 4E, 5E, Supplementary files 4–5). Furthermore, intestinal permeability in BB and VB mice showed a significant decrease compared to that in BTBR and VPA mice (Figs. 4F, 5F). This suggests that *C. butyricum* can contribute to improvements in intestinal barrier function in both ASD mouse models.

Furthermore, mice treated with *C. butyricum* exhibited increased expression of Trek1, claudin1, claudin3, and occludin proteins, as well as their corresponding levels of mRNA in the colon (Figs. 4G, H and 5G, H). This suggests that *C. butyricum* may enhance the integrity of tight junctions in the intestinal epithelium, further supporting the improvement in intestinal barrier function. Parallelly, the expression levels of pro-inflammatory cytokines (IL-6, TNF-α, and IFN-γ) in the colon of mice BB and VB were significantly lower compared to those of BTBR and VPA mice (Figs. 4I, 5I). These results suggest that the administration of *C. butyricum* is associated with increased fecal levels of *C. butyricum* and butyrate, improved intestinal barrier function, enhanced expression of tight junction proteins, decreased expression of histone deacetylases 1 (HDAC1), and reduced levels of pro-inflammatory cytokines in the colon of ASD mouse models.

### *Clostridium butyricum* modulates behavioral abmormalities in BTBR and VPA offspring

**Fig. 6:**
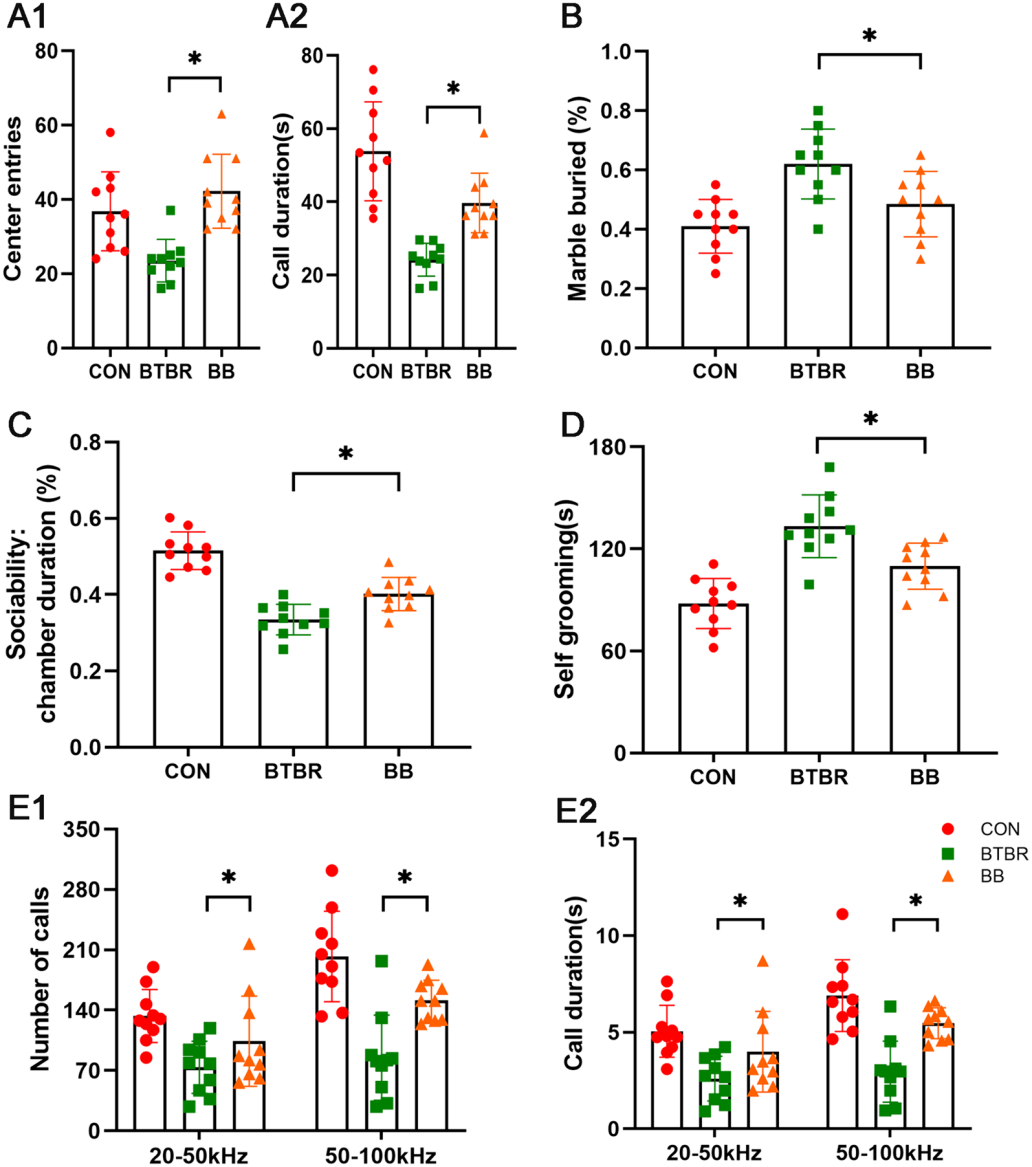
Behavioral abnormalities in BTBR offspring under *Clostridium butyricum* treatment. **A** The open field exploration performance of control, BTBR, and BB mice(A1: center entries; A2: call duration). **B** The marble buried of control, BTBR, and BB mice. **C** The sociability of control, BTBR, and BB mice. **D** The self grooming test of control, BTBR, and BB mice. **E1** The number of calls under different hertz of control, BTBR, and BB mice. **E2** The duration of calls under different hertz of control, BTBR, and BB mice. Significant differences are indicated by *p < 0.05 vs. BTBR group

**Fig. 7:**
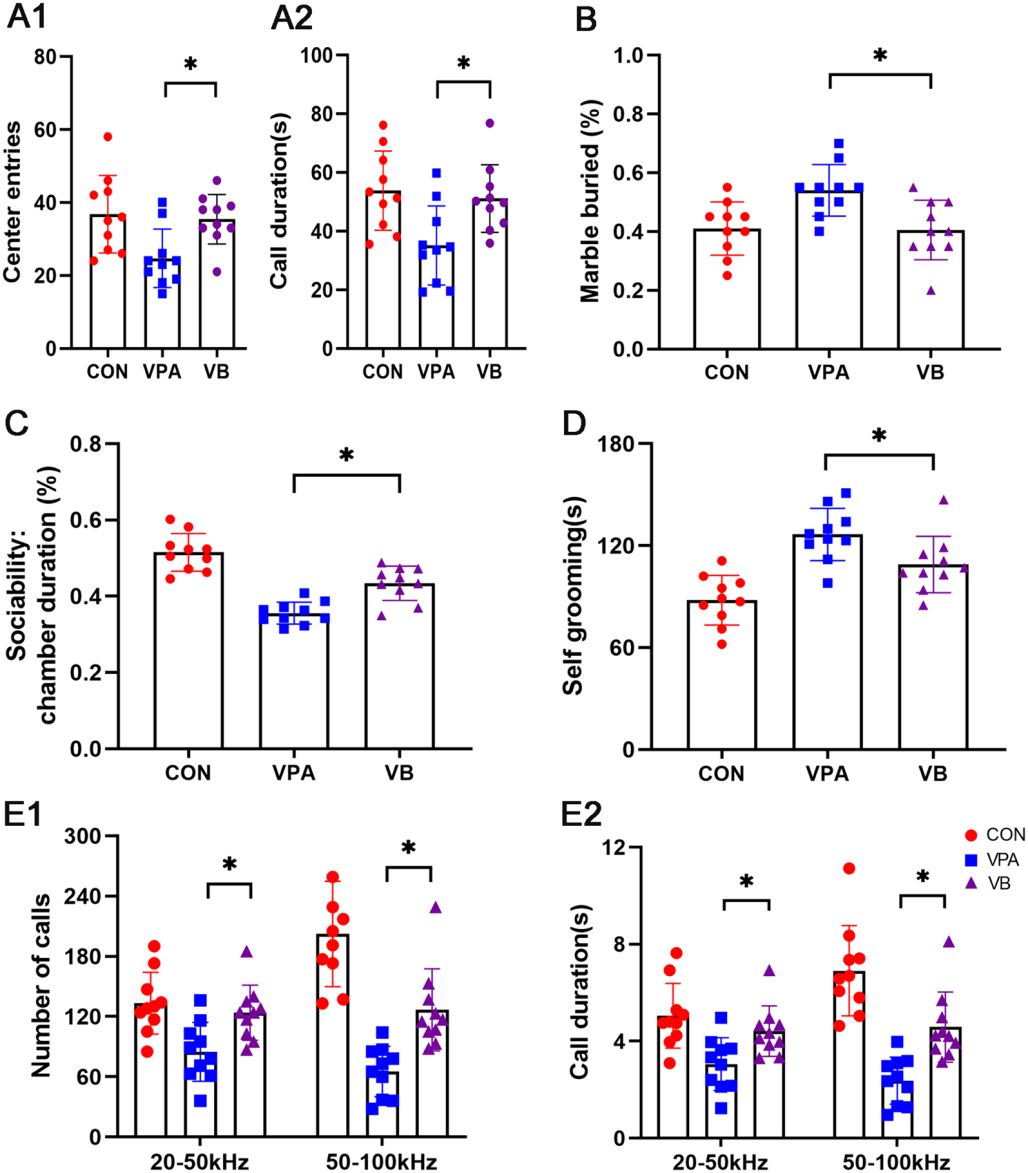
Behavioral abnormalities in VPA offspring under *Clostridium butyricum* treatment. **A** The open field exploration performance of control, VPA, and VB mice (A1: center entries; A2: call duration). **B** The marble buried of control, VPA, and VB mice. **C** The sociability of control, VPA, and VB mice. **D** The self grooming test of control, VPA, and VB mice. **E1** The number of calls under different hertz of control, VPA, and VB mice. **E2** The duration of calls under different hertz of control, VPA, and VB mice. Significant differences are indicated by *p < 0.05 vs. VPA group

We then investigated the behavioral performance of mice treated with *C. butyricum* (BB and VB). A similar pattern was observed in both ASD mice models, in which mice treated with *C. butyricum* exhibited increased center entries, center duration and chamber duration (novel mouse), decreased marble buried ratio (Fig. S7A), decreased time on self grooming and increased number of calls and call duration in both 20–50kHz and 50–100kHz (Figs. 6, 7, Fig. S7B). Our results suggested that *C. butyricum* treatment can reduce the autism-like behavior performances of mice in both models of ASD mice.

### *Clostridium butyricum* modulates intestinal barrier function through Trek1 in BTBR and VPA offspring

**Fig. 8:**
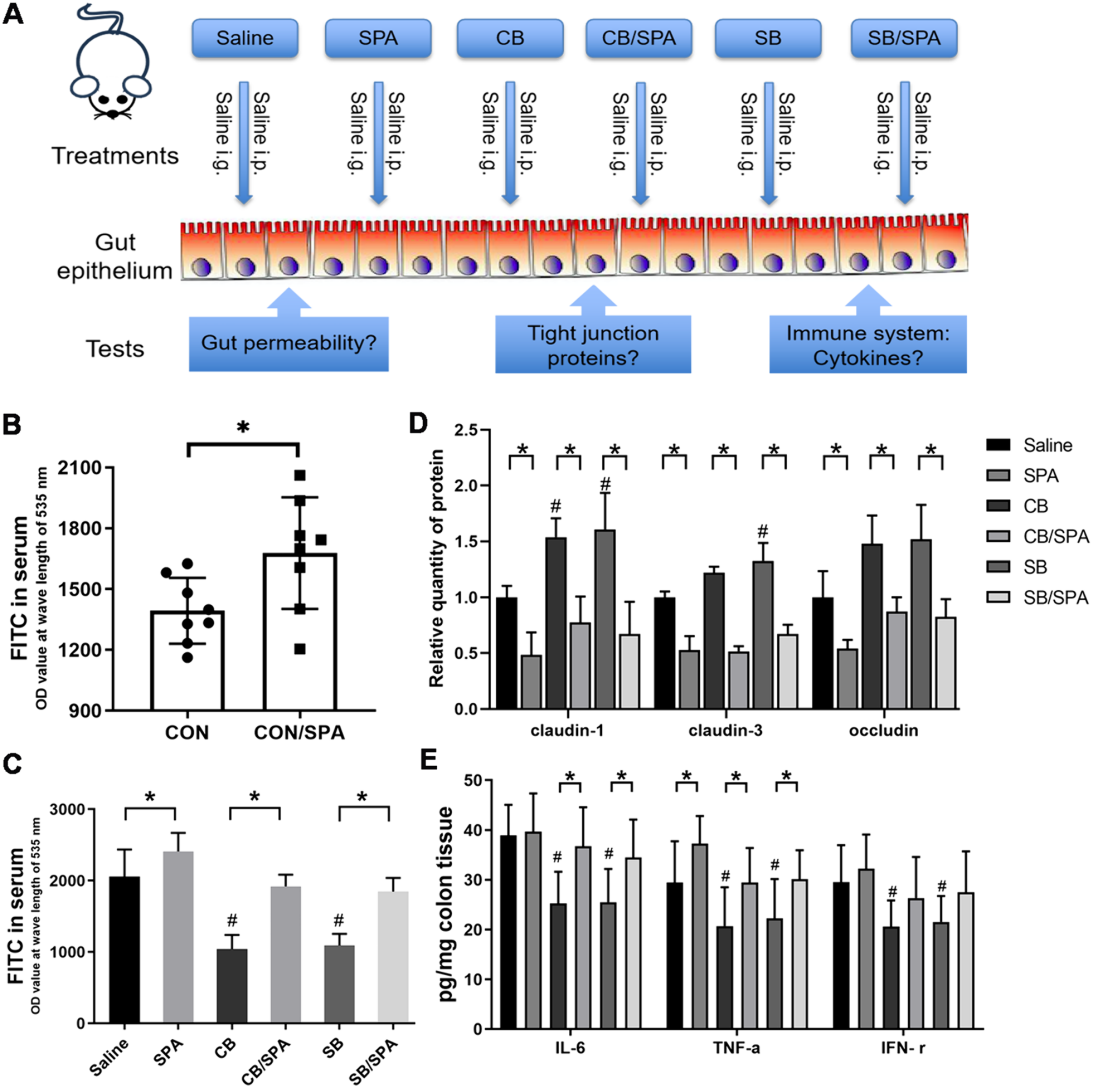
*Clostridium butyricum* modulates intestinal barrier function through Trek1 in BTBR and VPA offspring. **A** The experimental design outlook. **B**, **C** FITC in serum of different mice groups. **D** The relative quantity of protein claudin-1, claudin-3, and claudin of different mice groups. **E** The content of pro-inflammatory cytokines of different mice groups. Significant differences are indicated by *p < 0.05 and ^#^*p* < 0.05 vs. Saline group

To explore the potential target of *C.butyricum* or sodium butyrate on the intestinal epithelial barrier in ASD mice, we tested whether Spadin (Trek1 blocker) impacts tight junction proteins, pro-inflammatory cytokines, and intestinal permeability (Fig. 8A). We found that the administration of Spadin showed distinct effects on intestinal permeability and related markers in different mouse models. In C57bl/6J mice, Spadin administration can lead to an increase in intestinal permeability, indicating its disruptive impact on the intestinal barrier (Fig. 8B). However, in BTBR mice, Spadin administration reversed the reduced intestinal permeability induced by *C. butyricum* and sodium butyrate (Fig. 8C). This suggests that Spadin has the potential to restore barrier function in these mice. Furthermore, Spadin administration reversed the higher levels of tight junction proteins and lower concentrations of pro-inflammatory cytokines IL-6 and TNF-α observed in the colon of BTBR mice treated with *C. butyricum* and sodium butyrate (Fig. 8D, E). These findings suggest that Spadin may modulate the expression of TJ protein and possess anti-inflammatory properties, counteracting the effects of *C. butyricum* and sodium butyrate on intestinal barrier function and inflammation.

## Discussion

The findings of this study provide valuable information on the possible involvement of intestinal barrier dysfunction and altered gut microbiota in the models of ASD in BTBR and VPA mice. The validation of ASD models was characterized by the core behavioral deficits, including impaired communication and social interaction, due to the lack of a definite known diagnostic marker and the complexity of pathogenesis [40]. Basically, there are two types of animal models for ASD: genetically manipulated and environmentally induced, by prenatal exposure to certain chemicals or inflammation. BTBRT^+^Itpr3^tf^/J mice, an inbred strain with stable genetic background, exhibit behavioral abnormalities of ASD. Moreover, it is reported that the BTBR mouse severely reduces hippocampal commissure and absent corpus callosum [41]. VPA, a widely used antiepileptic drug, increases the risk of ASD while used in the first trimester of pregnancy [42]. The VPA mouse model displays autistic-like behaviors as well as neuroanatomical and cellular changes similar to autistic patients, including enhanced synaptic plasticity of prefrontal cortex [43]. In our study, the dysbiosis of the intestinal microbiota and the alteration of gut permeability observed in the BTBR and VPA offspring are consistent with the altered composition and function of the intestinal microbiota reported in ASD individuals [44].

Several studies discovered intestinal microbiota dysbiosis of the gut microbiota in ASD subjects [5, 45, 46]. We found that BTBR mice have a very distinct gut microbiota profile (including an increase in Firmicutes / Bacteroidetes ratio) with low diversity. Consistent with these observations, an increase in Firmicutes / Bacteroidetes ratio, which is associated with several inflammatory conditions [47], has been reported in ASD patients [48, 49]. However, we did not find an increased Firmicutes / Bacteroidetes ratio in VPA mice. In particular, we observed that the relative abundance of the order *Lactobacillales* was significantly increased in the gut microbiota of both BTBR and VPA mice with respect to that of controls while the relative abundance of *Erysipelotrichales* and *Spirochaetales* was significantly reduced in these mice. Regarding the role of *Lactobacillus* in the pathogenesis of ASD, the results of previous studies are not consistent. Stratiet al. demonstrated that *Lactobacillus* was enriched in the gut microbiota of autistic individuals [50], while Liu et al. found that *Lactobacillus plantarum* ameliorated opposition/defiance behaviors in boys with ASD [51]. Accordingly, more studies are needed to better clarify the role of *Lactobacillales*, *Erysipelotrichales,* and *Spirochaetales* in ASD. The lower species richness, diversity, and evenness observed in BTBR offspring, along with the distinct clustering of microbiota composition in both BTBR and VPA offspring, indicate significant deviations from the microbiota composition of control offspring. These alterations in the structure of the microbial community, characterized by shifts in the abundance of specific bacterial taxa, further support the notion of a disrupted gut microbiota in ASD.

The fact that *C. butyricum* treatment decreased *Verrucomicrobiota* in the BTBR model but increased it in the VPA model suggests that the underlying gut microbiome alterations and host-microbe interactions may differ between these two ASD animal models. This could be due to inherent differences in the genetic and environmental factors that contribute to ASD pathogenesis in each model [52]. The baseline gut microbiome composition and metabolic profiles may vary between BTBR and VPA mice, leading to divergent responses to the *C. butyricum* intervention. Moreover, the nature and extent of gut barrier dysfunction and mucin dysregulation may differ between the two ASD models, resulting in distinct effects on *Verrucomicrobiota* when *C. butyricum* is administered. Understanding these model-specific differences in microbiome responses to *C. butyricum* may help inform the development of more personalized, microbiome-targeted therapies for ASD patients. Exploring the underlying factors driving these divergent effects could lead to a better understanding of the heterogeneity in gut microbiome alterations associated with ASD [17].

The differences in short-chain fatty acid levels between ASD mice and control mice were significant. SCFA, such as acetic acid, propionic acid, butyric acid, and valeric acid, are important microbial metabolites that play a vital role in the microbiome-gut-brain axis. The altered SCFA profiles observed in the BTBR and VPA offsprings, particularly the lower levels of butyric acid in both the BTBR and VPA offsprings, suggest potential disruptions in SCFA production and signaling in these models of ASD. Furthermore, our previous study identified decreased abundances of key butyrate-producing taxa and reduced fecal butyric acid in autistic children [32], suggesting butyric acid plays an important role in the pathogenesis of ASD. The administration of *Clostridium butyricum*, a probiotic bacterium known for its beneficial effects on gut health [53], revealed promising results in modulating intestinal barrier function and behavior in the BTBR and VPA offspring. In addition, the behavioral improvements observed in mice treated with *C. butyricum,* such as increased exploratory behavior and reduced repetitive behaviors, suggest a potential link between gut microbiota modulation and behavioral outcomes in ASD. These findings align with the growing recognition of the gut-brain axis and the influence of the gut microbiota on neurodevelopment and behavior.

The Trek1 channel, a member of the two-pore domain potassium channel family, plays a role in regulating intestinal barrier function and gut homeostasis [54]. We found decreased expression of Trek1 in the colon of BTBR and VPA offspring, suggesting a potential role for this channel in maintaining intestinal barrier integrity. These findings are consistent with previous studies that have implicated Trek1 in the regulation of gut homeostasis and barrier function [55, 56]. The increased expression of Trek1 and the tight junction proteins that form barriers, as well as the decreased expression of pro-inflammatory cytokines, in the colon of mice treated with *C. butyricum* indicates a potential role for this probiotic in the restoration of intestinal barrier integrity and the reduction of inflammation. Previous studies have shown that alterations in the composition of the gut microbiota, including an overgrowth of *Clostridium* species, are associated with ASD. Furthermore, the use of a Trek1 blocker, Spadin, demonstrated that the beneficial effects of *C. butyricum* and sodium butyrate on intestinal barrier function in BTBR mice were mediated, at least in part, through the Trek1 channel. This highlights the potential therapeutic target of Trek1 in improving the integrity of the gut barrier in ASD. Thus, our study herein fills the gap on the specific mechanisms by which the gut microbiota, including *C. butyricum*, influence ASD-related behaviors and GI dysfunction.

## Conclusions

**Fig. 9:**
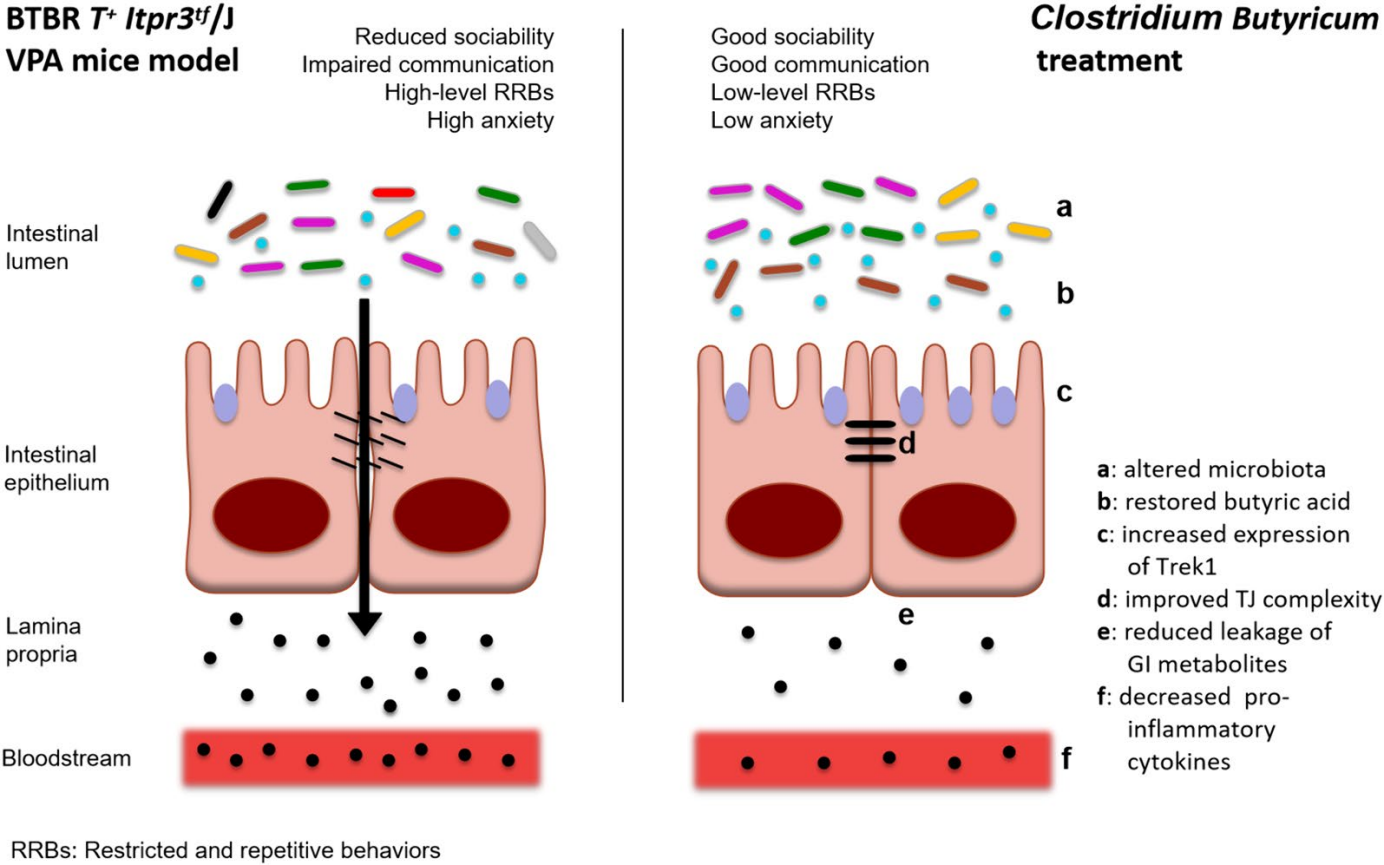
The proposed mechanisms of *Clostridium butyricum* treatment in ASD mice

The present study provides compelling evidence supporting the involvement of intestinal barrier dysfunction and intestinal microbiota dysbiosis in ASD. The findings suggest that interventions targeting the gut microbiota, such as probiotics *C. butyricum*, hold promise for improving the function of the gut barrier and improving the behavioral abnormalities associated with ASD (Fig. 9). More research is needed to elucidate the precise mechanisms underlying these effects and explore the translational potential of these findings for patients with ASD.

### Supplementary Information

Supplementary material 1. Supplementary methods, supplementary table and 7 supplementary figures.

Supplementary material 2. The differentially abundant bacterial taxa at the species level between control and BTBR groups.

Supplementary material 3. The differentially abundant bacterial taxa at the species level between control and VPA groups.

Supplementary material 4. The differentially abundant bacterial taxa at the species level between BTBR and BB groups.

Supplementary material 5. The differentially abundant bacterial taxa at the species level between VPA and VB groups.

## Data Availability

16S rRNA gene sequencing data are available from NCBI (PRJNA1070713). The other datasets used during the current study are available from the corresponding author upon reasonable request.

## Abbreviations

ASD: Autism spectrum disorder
GI: Gastrointestinal
BTBR: BTBR T^+^ Itpr3^tf^/J
VPA: Valproic acid
CNS: Central nervous system
B6J: C57B/6J
OTUs: Operational taxonomic units
SI: Small intestine
SCFA: Short-chain fatty acids
HPLC: High-performance liquid chromatography system
ELISA: Enzyme-linked immunosorbent assay
USV: Ultrasonic vocalization
TJ: Tight junction
Trek1: TWIK-related potassium channel-1
ZO-1: Zonula occludens-1
PCA: Principal component analysis
LDA: Linear discriminant analysis
LEfSe: Linear discriminant effect size
HDAC1: Histone deacetylases 1
