# Supplementary material for: Clostridium butyricum regulates intestinal barrier function via trek1 to improve behavioral abnormalities in mice with autism spectrum disorder
Source: Cell Biosci. 2024 Jul 21;14:95. doi: 10.1186/s13578-024-01278-6 (PMC11265103; doi:10.1186/s13578-024-01278-6)
Supplement: Supplementary file 1 — Supplementary material 1. Supplementary methods, supplementary table and 7 supplementary figures. [file 13578_2024_1278_MOESM1_ESM.docx]

**Supplementary information**

Supplementary information includes supplementary methods, supplementary table and 7 supplementary figures.

**Supplementary methods**

**Study design treatment and groups**

B6J and BTBR mice were paired overnight. Pregnant B6J(age 8-10 weeks) were injected i.p. on E12.5 with saline or 600 mg/kg VPA (Sigma Aldrich) according to the methods described by Almeida^1^. At the age of 3 weeks, ten male offspring of these three groups were sacrificed for testing the intestinal barrier function (randomly selected, at most 2 offspring from the same litter).

**Behavioral test**

**Open field exploration**

Mice were placed in a 50 × 50 cm white Plexiglas box for 10min. The traveled distance, the number of center entries and the duration of time spent in the center arena (17×17 cm) were recorded and analyzed using Any-maze software (Stoelting Co., USA)^2^.

**Marble burying test**

Marble burying was modified based on methods described by Thomas et al.^3^ and Malkova et al.^4^. Mice were habituated for 10 min to a clean cage (42.5× 26.5 × 19 cm) containing a 5 cm layer of chipped cedar wood bedding and then placed back into the home cage. Twenty black glass marbles (15 mm diameter) were aligned equidistantly 4 × 5 in the testing cage. Mice were returned to the testing cage and the number of marbles buried (>2/3 marble covered by bedding material) in 10 min was recorded.

**Social interaction test**

Mice were tested for sociability using a 60×40 cm Plexiglas three-chambered apparatus as described by Yang et al.^5^. Briefly, testing mice were habituated for 10 min to the center of the apparatus. To evaluate preference for either of the side chambers, the testing mice were allowed 10 min to explore the whole apparatus with clear interaction cylinders (14cm height, 5.5 cm diameter, bars spaced 1 cm apart) in each of the side chambers. We confirmed that there was no bias for every mouse. Sociability was tested in the following 10 min session, where the testing mouse were allowed to explore a novel same-sex mouse in one clear interaction cylinder versus a novel object (green sticky ball) in the other clear interaction cylinder of the opposite side chamber. Any-maze software was used to record and measure the duration spent in the each of the three chambers.

**Adult** **ultrasonic vocalizations**

Adult ultrasonic vocalizations (USVs) were conducted and analyzed as described by Malkova et al.^4^ andScattoni et al.^6^. Adult males were housed singly for one week before testing and exposed for 20 min to an unfamiliar adult female mouse every day for five days prior to testing to provide a standardized history of sexual experience. On testing day, mice were habituated to a MDF sound-attenuating cubicle for 10 minutes. USVs were recorded for 3 min using a USV detector (MED Associates, USA) in the presence of a novel age-matched female in the estrous phase. The number of calls and total call duration were recorded and analyzed using Med USV application software.

**Self-grooming test**

Self-grooming (NIMH)

In addition to the grooming measures scored during the juvenile play

session within the NoldusPhenotyper (illustrated in Videos S3 and

S4), a separate set of male B6 (n ¼ 10) and BTBR (n ¼ 10) mice were

scored for spontaneous grooming behaviors when placed individually

in a clean, empty mouse cage without bedding. Each mouse was

given a 10-min habituation period in the empty cage and then rated for

10 min for cumulative time spent grooming all body regions. The

investigator sat approximately 2 m from the test cage and recorded

cumulative time spent in grooming with a stopwatch. The same mice

were tested at 18, 28, 38 and 60 days of age.

Self-grooming (NIMH)

In addition to the grooming measures scored during the juvenile play

session within the NoldusPhenotyper (illustrated in Videos S3 and

S4), a separate set of male B6 (n ¼ 10) and BTBR (n ¼ 10) mice were

scored for spontaneous grooming behaviors when placed individually

in a clean, empty mouse cage without bedding. Each mouse was

given a 10-min habituation period in the empty cage and then rated for

10 min for cumulative time spent grooming all body regions. The

investigator sat approximately 2 m from the test cage and recorded

cumulative time spent in grooming with a stopwatch. The same mice

were tested at 18, 28, 38 and 60 days of age.

This test was conducted as described by Mcfarlane et al.^7^. Mice were habituated for 10min in an empty cage without bedding (28 × 17 × 12 cm). Afterwards, self-grooming was measured for a further 10 min. The investigator sat approximately 1.5 m away from the test cage and recorded cumulative time spent in grooming all body region with a silent stopwatch^7^.

**Supplementary references**

1. Almeida, L. E. F., Roby, C. D. & Krueger, B. K. Increased BDNF expression in fetal brain in the valproic acid model of autism. *Mol. Cell. Neurosci.***59**, 57–62 (2014).

2. Hsiao, E. Y. *et al.* Microbiota modulate behavioral and physiological abnormalities associated with neurodevelopmental disorders. *Cell***155**, 1451–1463 (2013).

3. Thomas, A. *et al.* Marble burying reflects a repetitive and perseverative behavior more than novelty-induced anxiety. *Psychopharmacology (Berl.)***204**, 361–373 (2009).

4. Malkova, N. V., Yu, C. Z., Hsiao, E. Y., Moore, M. J. & Patterson, P. H. Maternal immune activation yields offspring displaying mouse versions of the three core symptoms of autism. *Brain. Behav. Immun.***26**, 607–616 (2012).

5. Yang, M., Silverman, J. L. & Crawley, J. N. Automated three-chambered social approach task for mice. *Curr. Protoc. Neurosci.***Chapter 8**, Unit 8.26 (2011).

6. Scattoni, M. L., Ricceri, L. & Crawley, J. N. Unusual repertoire of vocalizations in adult BTBR T+tf/J mice during three types of social encounters. *Genes Brain Behav.***10**, 44–56 (2011).

7. McFarlane, H. G. *et al.*Autism-like behavioral phenotypes in BTBR T+tf/J mice. *Genes Brain Behav.***7**, 152–163 (2008).

**Supplementary Table**

**Table S1 Sequences of primers used in the study**

| Gene | Forward primer (5'->3') | Reverse primer (5'->3') | Length(bp) |
| --- | --- | --- | --- |
| HDAC1 | CAGAACACTAACGAGTAC | GGAGCAGATGGAGATGCG | 171bp |
| TREK-1 | CTACGTGGCAGGTGGATCAG | CATGCGCTCTGAACTCTCCC | 173bp |
| Claudin1 | ACATTAAGTATGAGTTTGGC | ACCCAGCCTTGCTCTCATTC | 117bp |
| Claudin3 | GTACAAGACGAGACGGCCAA | CGTACAACCCAGCTCCCATC | 175bp |
| Occludin | GTCCTCCTGGCTCAGTTGAA | AGATAAGCGAACCTTGGCGG | 103bp |
| IL-6 | GCCTTCTTGGGACTGATGCT | TGCCATTGCACAACTCTTTTCT | 181bp |
| TNF-α | AGGCACTCCCCCAAAAGATG | GCCATTTGGGAACTTCTCAT | 158bp |
| IFN-γ | ACTGTGATTGCGGGGTTG | TCACTGCAGCTCTGAAT | 118bp |
| GAPDH | GTGAGGCCGGTGCTGAGTAT | GTGCAGGATGCATTGCTGAC | 196bp |

**Supplementary Figures**

**A：**


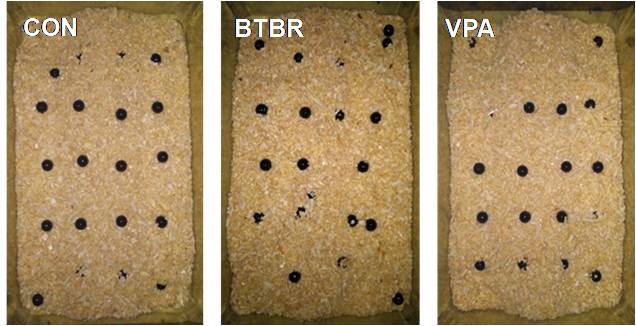


**B:**


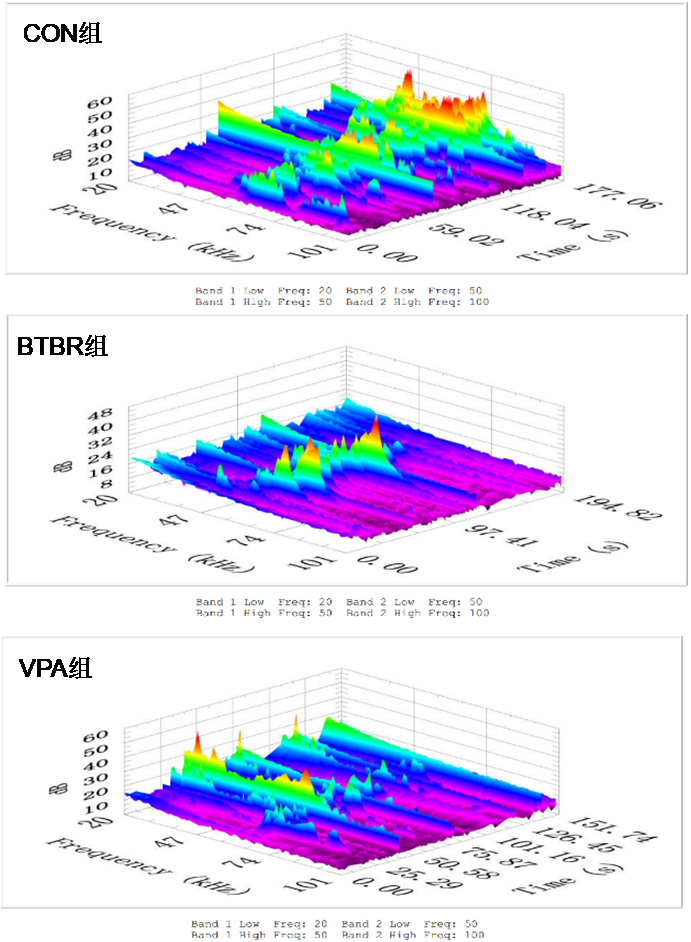


**Figure S1. BTBR and VPA offspring display ASD-Related Behavioral Abnormalities**

**A**. Marble burying test **B**. Ultrasonic vocalizations.

**
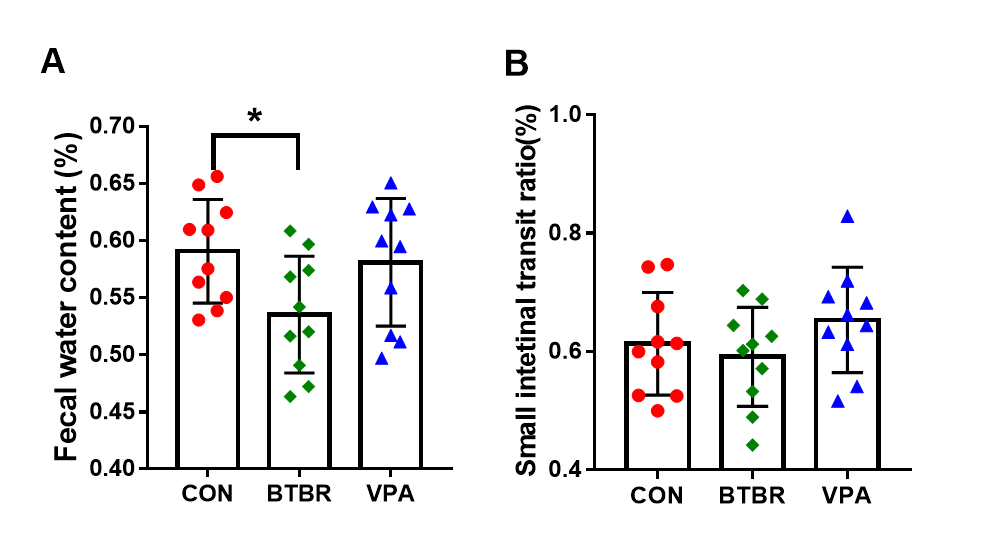
**

**Figure S2. BTBR offspring show decreased fecal water content**

**A**. The fecal water content was decreased in BTBR offspring. **B**. No significant differences were found between BTBR/VPA offspring and controls regarding the SI transit ratio.

**p*<0.05. ns: not statically significant. n=10/group.

**
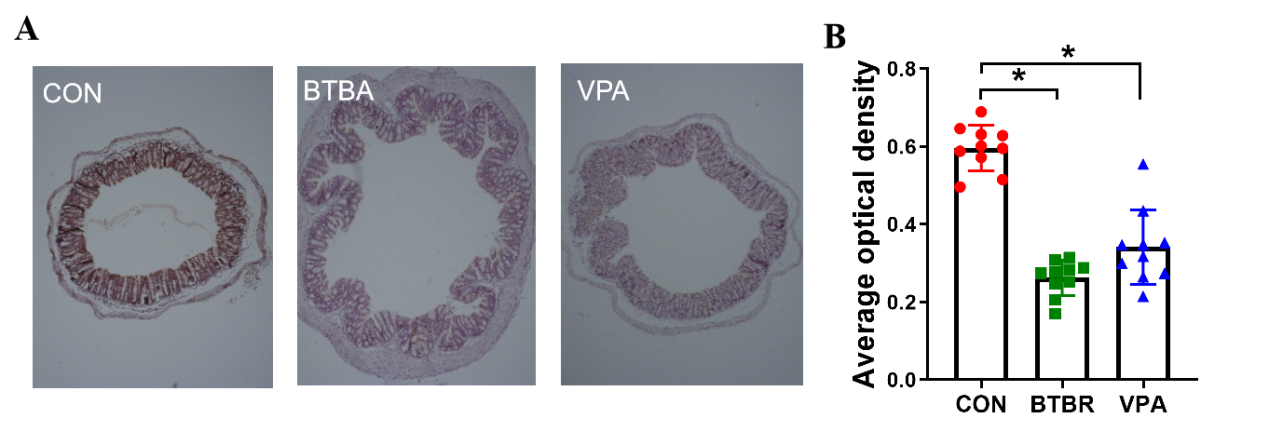
**

**Figure S3. Immunohistochemistry staining for Trek1**

A. The expression of Trek1 using IHC staining(50X). B. The quantification of Trek1 was performed using optical density analysis with ImageJ software. **p*<0.05. n=10/group.

*
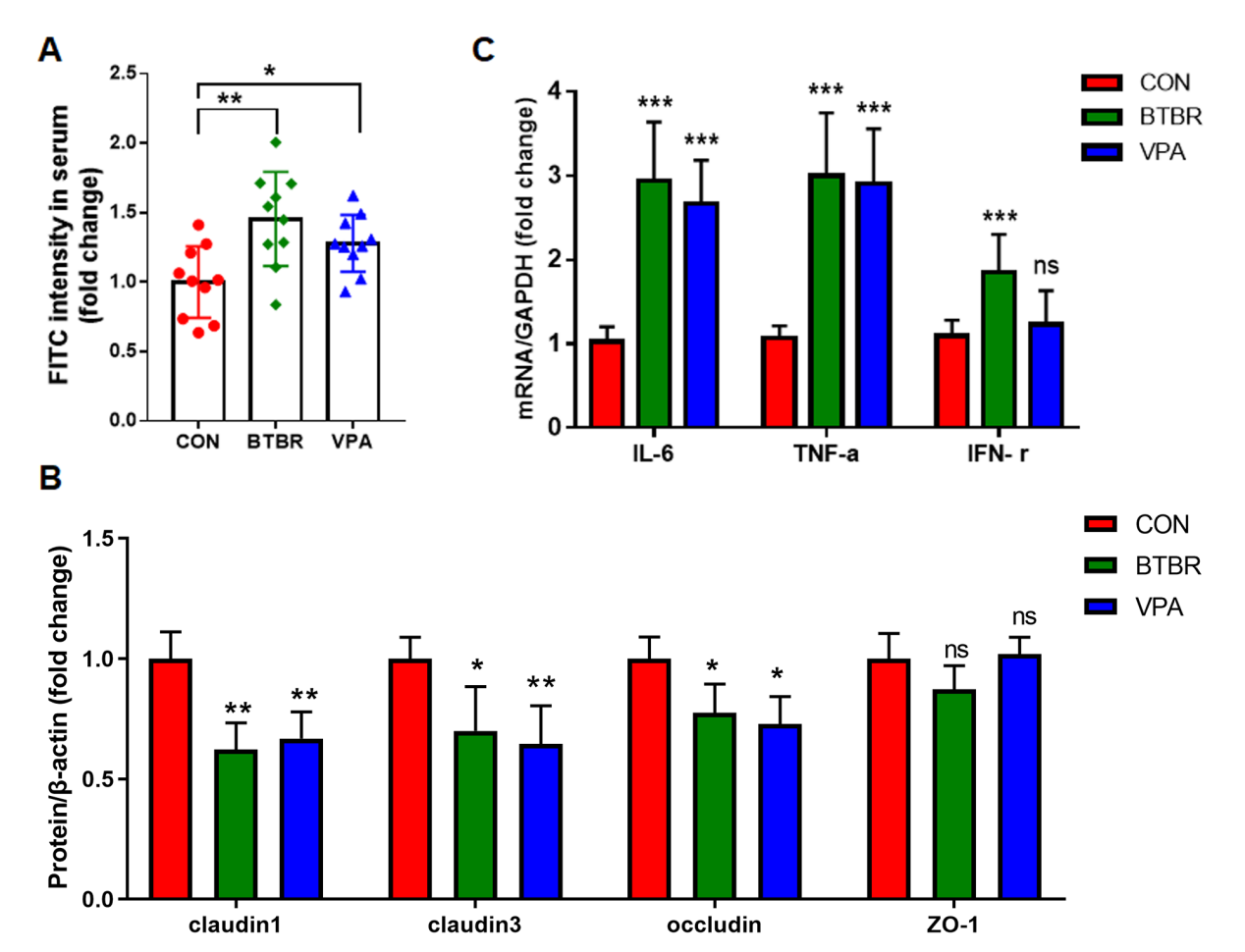
*

**Figure S4. Intestinal barrier dysfunction in 3-week-old offspring**

**A**. Intestinal permeability assay, measuring translocation of 4-kDa FITC-dextran from the intestinal lumen into the blood. **B**. Colon protein levels of tight junction components relative to β-actin. **C**. Colon mRNA expression of cytokines relative to GAPDH.

Data are normalized to controls. **p*<0.05; ***p*<0.01; ****p*<0.001; ns: not statically significant. n=10/group.


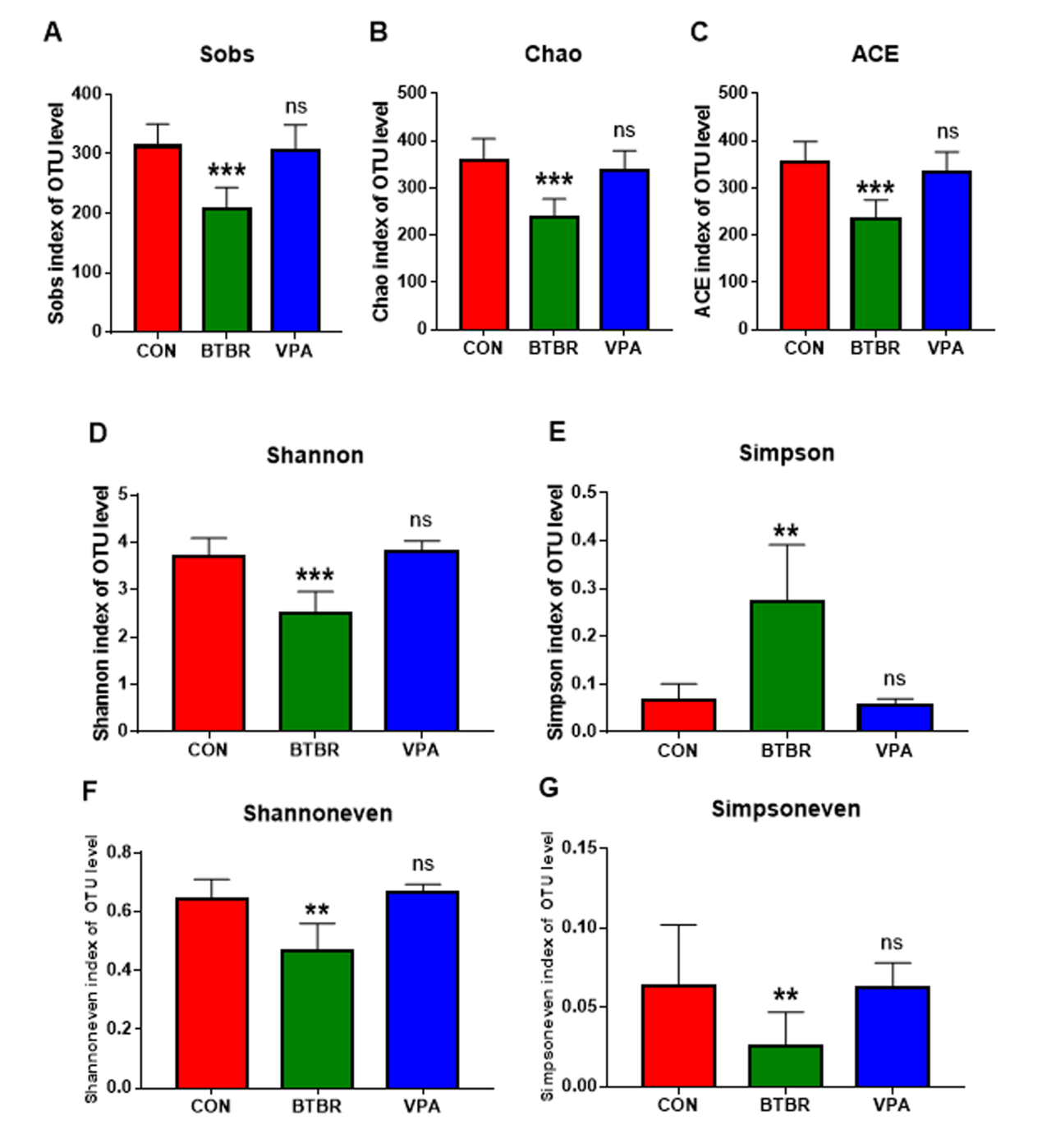


**Figure S5. BTBR offspring show decreased microbial alpha diversity**

Alpha diversity based on the Sobs (**A**), Chao (**B**), ACE (**C**), Shannon (**D**), Simpson(**E**), Shannoneven(**F**) and Simpsoneven (**G**) indices of the OTU level.

***p*<0.01; ****p*<0.001; ns: not statically significant. n=8/group.

**
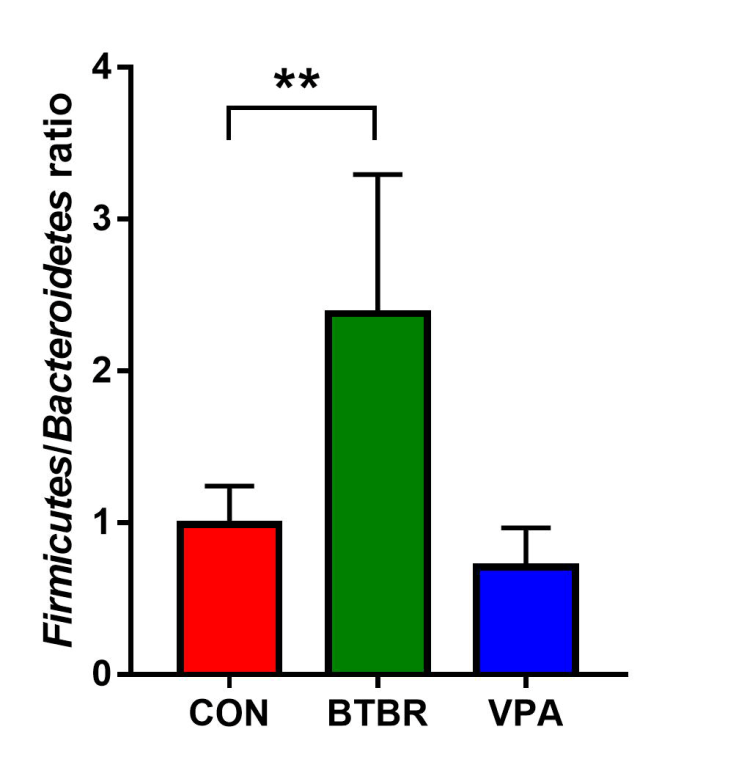
**

**Figure S6. BTBR offspring show an increased** **Firmicutes/Bacteroidetes ratio**

***p*<0.01. n=8/group.

**A:**

CON BTBR BB VPA VB


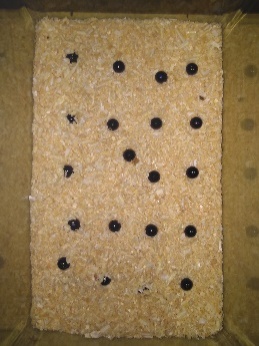

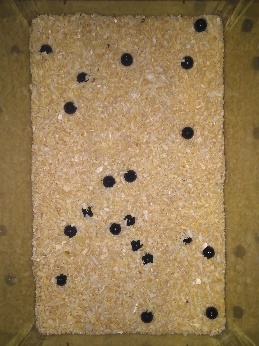

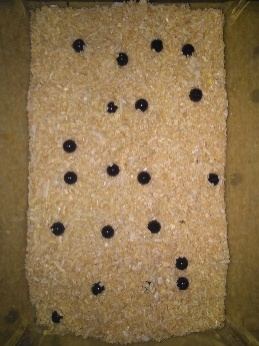

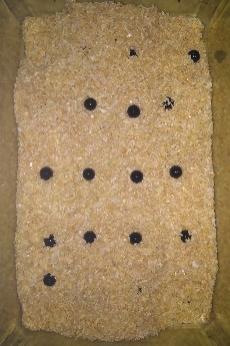

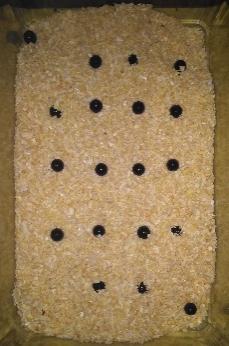


**B:**

CON


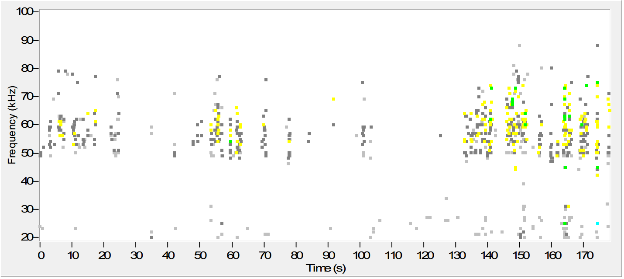

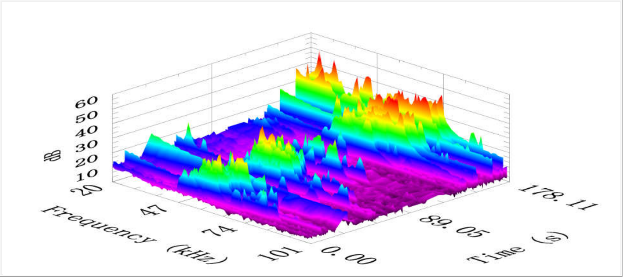


BTBR


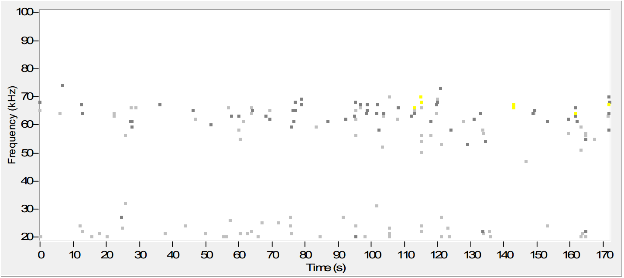

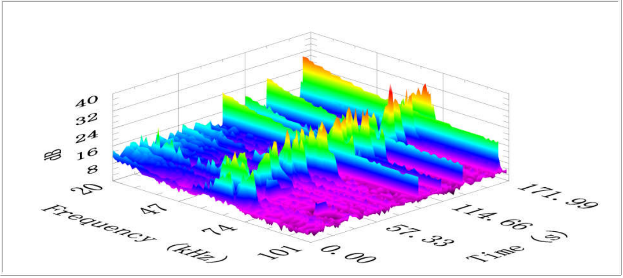


BB


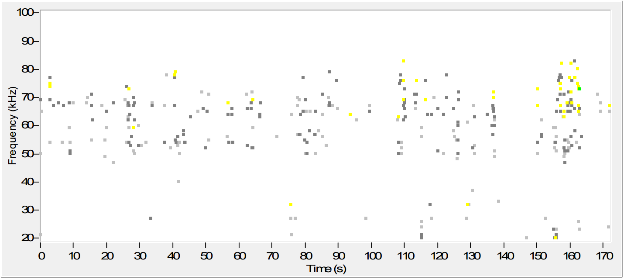

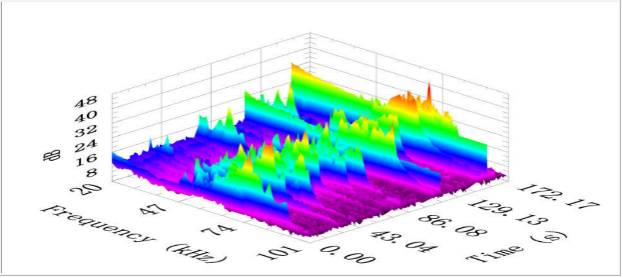


VPA


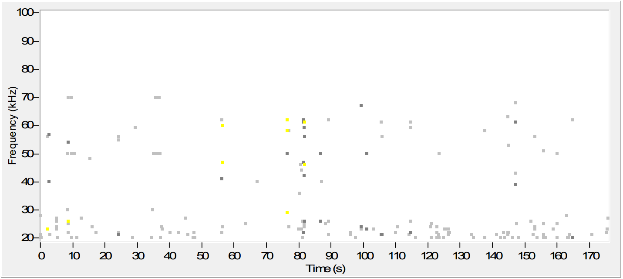

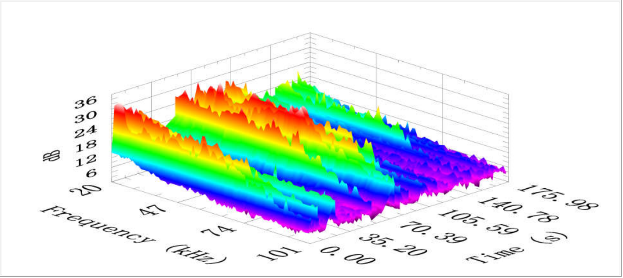


VB


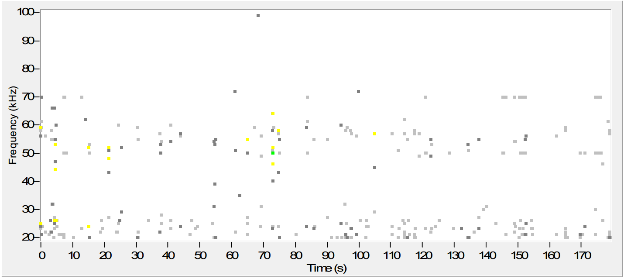

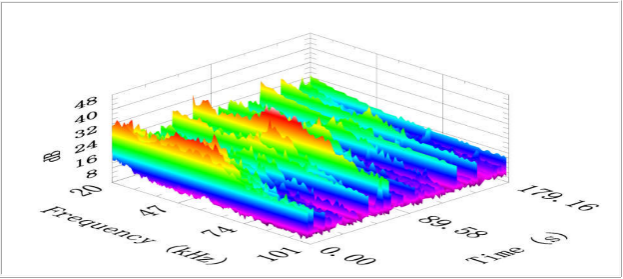


**Figure S7. *C. butyricum* modulates ASD behavioral abmormalities in BTBR and VPA offspring**

1. Marble burying test **B**. Adult ultrasonic vocalizations in a social encounter with a female.
